# Query expansion using MeSH terms for dataset retrieval: OHSU at the bioCADDIE 2016 dataset retrieval challenge

**DOI:** 10.1093/database/bax065

**Published:** 2017-10-20

**Authors:** Theodore B Wright, David Ball, William Hersh

**Affiliations:** 1 Department of Medical Informatics & Clinical Epidemiology, School of Medicine, Oregon Health & Science University, 5th Floor, Biomedical Information Communication Center (BICC) 3181 S.W. Sam Jackson Park Rd., Portland, OR 97239, USA

## Abstract

Scientific data are being generated at an ever-increasing rate. The Biomedical and Healthcare Data Discovery Index Ecosystem (bioCADDIE) is an NIH-funded Data Discovery Index that aims to provide a platform for researchers to locate, retrieve, and share research datasets. The bioCADDIE 2016 Dataset Retrieval Challenge was held to identify the most effective dataset retrieval methods. We aimed to assess the value of Medical Subject Heading (MeSH) term-based query expansion to improve retrieval. Our system, based on the open-source search engine, Elasticsearch, expands queries by identifying synonyms from the MeSH vocabulary and adding these to the original query. The number and relative weighting of MeSH terms is variable. The top 1000 search results for the 15 challenge queries were submitted for evaluation. After the challenge, we performed additional runs to determine the optimal number of MeSH terms and weighting. Our best overall score used five MeSH terms with a 1:5 terms:words weighting ratio, achieving an inferred normalized distributed cumulative gain (infNDCG) of 0.445, which was the third highest score among the 10 research groups who participated in the challenge. Further testing revealed our initial combination of MeSH terms and weighting yielded the best overall performance. Scores varied considerably between queries as well as with different variations of MeSH terms and weights. Query expansion using MeSH terms can enhance search relevance of biomedical datasets. High variability between queries and system variables suggest room for improvement and directions for further research.

**Database URL:** https://biocaddie.org/benchmark-data

## Introduction

Biomedicine is seeing substantial growth in research generating large sets of data (1,2). Biomedical datasets are highly variable and can range from collections of genomic sequences to large clinical data repositories from the electronic health record (EHR) and other sources. Numerous datasets are now available for researchers in various repositories such as the National Center for Biotechnology Information’s (NCBI) Gene Expression Omnibus (GEO) (3), Reference Sequence (RefSeq) (4), the database of genotypes and phenotypes (dbGap) (5), and many more. Recognizing the need to facilitate integration of data from various sources, the National Institute of Health (NIH) Big Data to Knowledge (BD2K) initiative funded development of Biomedical and Healthcare Data Discovery Index Ecosystem (bioCADDIE), which aims to provide a platform to retrieve relevant metadata about entire datasets.

Shared tasks enable researchers from many different institutions to work together at solving a common scientific challenge. In biomedicine, shared tasks such as bioASQ (6), JNLPBA (7), bioNLP (8) and BioCreative (9) have contributed significantly to the field. Shared tasks have also played a considerable role in the advancement of Information Retrieval (IR) methods. One such shared task is the National Institute of Standards of Technology’s (NIST) yearly challenge evaluation, the Text REtrieval Conference (TREC, http://nist.trec.gov) (10–12). During TREC challenges, participants are provided test collections and topics to configure an IR system and provide search output. Results are then pooled from all participating research groups and judged for relevance (13).

To improve the existing prototype search engine and determine the best approaches for indexing and retrieving records in bioCADDIE, the organizers held a challenge evaluation based on the TREC format. In the bioCADDIE 2016 Dataset Retrieval Challenge, participants were provided a database with sample queries and were tasked to develop a high-performance IR system. For a full description of the bioCADDIE 2016 challenge, including details of the shared task, test queries and links to datasets please see the overview paper (14).

Query expansion is a collection of methods used to improve search results through reformulation of the original search query, often with the addition and re-weighting of related terms. The technique is widely used in the biomedical domain and has yielded positive results in many IR tasks (15–18). Manual query expansion depends on user input to help select relevant terms (19), while automatic query expansion is performed entirely by the IR system with no additional user intervention. Query expansion techniques can vary with the determination of a number of variables involved in the process – the selection of related terms, the number of terms to add, and re-weighting of the new terms can all have a significant impact on the effects of query expansion (20).

The terms identified through query expansion can be lexically related such as in a thesaurus or vocabulary system, or statistically related such as in co-occurrence in a document collection. Lexically related terms can link terms across different vocabulary systems, e.g. a laymen’s term like ‘cancer’ to a more clinical term like ‘carcinoma’. Statistically related terms can help reduce the ambiguity of a single query term by providing contextual information (21). Query expansion using lexically-related terms from curated, domain-specific vocabularies such as the Medical Subject Headings (MeSH) of the National Library of Medicine (NLM), has been shown to have a positive effect in many biomedical literature retrieval tasks (17,19,22–25).

The best number of related terms and term weighting to use for query expansion, as well as techniques to do so, vary significantly among different methodologies. Abdulla et al. (16) evaluated four different methods of query expansion using the TREC 2006 and 2007 Genomic data-sets, and found the best number of terms ranged from 3 to 40 depending on the methodology used. Voorhees (21) evaluated query expansion using with lexically related terms using WordNet (26). She found that overly aggressive expansion with many terms reduced result quality, and that scores were marginally improved to a similar degree for all evaluated term weighting lighter than 1:1.

The bioCADDIE database, with its metadata about datasets, offers an opportunity to evaluate IR methods such as query expansion in a novel context. Our primary objective for the bioCADDIE 2016 dataset retrieval challenge was to enhance the baseline search functionality of an open source search platform by assessing automatic query expansion using lexically-related terms from the MeSH vocabulary and re-weighting of these terms. Because dataset retrieval is a relatively new undertaking, we opted to initially develop an approach that used a minimum amount of resources and allowed for fast and iterative prototyping.

After challenge completion, we performed additional analysis using the relevance judgments qrels file. This allowed us to further assess our methods as well as determine the optimal number of MeSH synonyms and weighting to use for the best system performance.

## Materials and methods

Our system is based on the open-source software, Elasticsearch v5.0.0, which acts as our core search engine. We used the Natural Language Toolkit (NLTK) module for Python (27) to perform basic query preprocessing, and the Biopython module (28) using the NLM’s Entrez service (29) to query the MeSH database and return relevant MeSH terms. All software settings were left as default except for the Java virtual machine heap size, which was changed to 1 g to better utilize available system memory. Table 1 provides a list of all software dependencies.

**Table 1.** Software dependencies

Role	Software
Indexing and Query Processor	Elasticsearch 5.0.0 (https://www.elastic.co/downloads/Elasticsearch)
Programming Language	Python 3.5 (https://www.python.org/downloads/)
Natural Language Processing (NLP) Framework	Natural Language Tool Kit (NLTK) (http://www.nltk.org/book/)
Python Application Programming Interface (API) to Search Service	Elasticsearch-py (https://Elasticsearch-py.readthedocs.io/en/master/)
	Elasticsearch_dsl (https://Elasticsearch-dsl.readthedocs.io/en/latest/)
API to Entrez	BioPython (http://biopython.org/wiki/Biopython)
Other Dependencies	Oracle Java Runtime Environment 1.8
Operating System	Microsoft Windows 10 64-bit

### Dataset and import

The 2016 bioCADDIE Search Retrieval Challenge database contains metadata records for 794 992 datasets from 19 different repositories. More details about the challenge database, including available metadata fields, can be found in the overview manuscript (30). To manage development with such a large database, we used a Python script to perform a fully-automatic import of all data. The Elasticsearch database is non-relational and stores data as independent documents. The structure of each document can be customized, but by default is derived from the source data. This allowed us to import the supplied JSON files directly into Elasticsearch as independent documents and, with a few exceptions, keep the underlying structured metadata fields intact. During the import process, the default Elasticsearch Standard Analyzer performed tokenization of all fields based on a Unicode standard algorithm (31) to build the search index. Sixty-three files were not successfully imported due to parsing errors.

### Search

**Figure 1.**
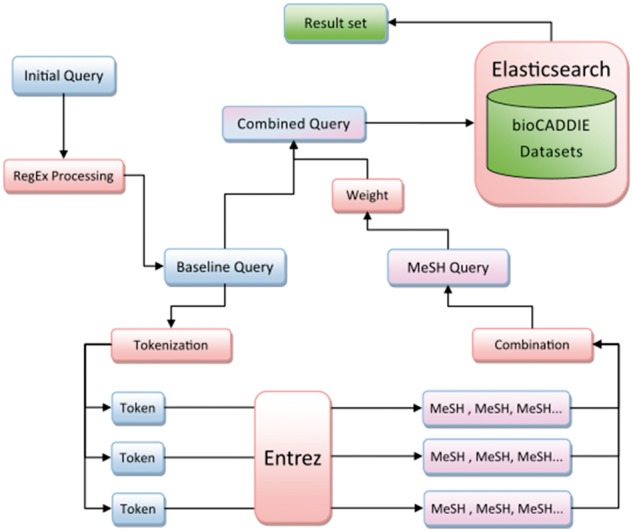
Method overview.

**Figure 2.**
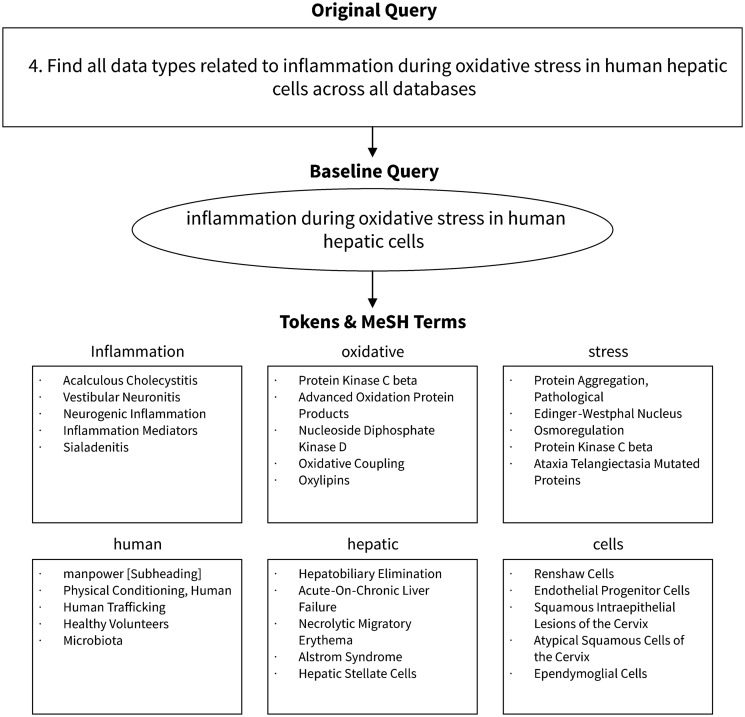
Example query processing.

Our search method is diagrammed in Figure 1, and Figure 2 provides an example of query processing and resulting MeSH terms using an actual query from the challenge. First, the query was normalized to lower case and common words and phrases such as ‘search for’ were removed using a regular expression. English stop words, as defined by the NLTK python module, were also removed. This preprocessing step resulted in the creation of our ‘baseline query’.

The baseline query was then tokenized into an array of individual tokens using NLTK’s tokenizer method. Each token was passed to the NLM’s Entrez service to find and fetch related terms from the MeSH database. Terms were returned sequentially, the order of which was determined by the Entrez service’s default settings.

The maximum MeSH terms returned for each token was capped at a variable number, however not all tokens would necessarily return the maximum number of results. All returned MeSH terms were combined as a single string to create an additional clause in the Elasticsearch query object. This entire ‘MeSH query’ was weighted against the baseline query via a ‘boost’ parameter, then combined with a ‘should’ clause using the Elasticsearch_dsl Python module. The cause defines terms as not required, but if found in results the results are considered more relevant (32). The final combined query was passed to Elasticsearch and results were output to a text file in the specified treceval formatting.

### Challenge submission

Each participating group was provided six training queries and 15 test queries. We did not incorporate the training queries into our methods. Results from up to five runs per participating group were pooled for relevance judgments. Relevance judgments for records retrieved for each query were performed by the challenge organizers (30). The treceval package was used to provide results to participating groups, with a focus on the parameters of inferred average precision (infAP), inferred normalized discounted cumulative gain (infNDCG), normalized discounted cumulative gain at 10 records (NDCG@10), precision at 10 documents for fully and partially relevant records (P@10 + partial), and precision at 10 documents for only fully relevant records (P@10-partial).

The challenge organizers chose infNDCG as the primary judgment for this shared task (14). Discounted Cumulative Gain (DCG) is an aggregation of recall and precision that is calculated based on each document returned in a ranked result batch. Relevance of documents as well as document ranking order is used in the calculation, with more relevant documents ranking higher yielding higher scores. Normalized DCG (NDCG) normalizes this score from 0 to 1.0 to facilitate comparison across different queries (13). Inferred scores, such as infNDCG, are used to estimate a result’s quality when the relevance of all documents is unable to be determined.

We submitted five runs for the initial challenge, as detailed in Table 2. Each run contained 1000 results per query. Run OHSU-1 used only the baseline query as input to Elasticsearch. Runs OHSU-2 through OHSU-4 combined the baseline query with the MeSH query as described above and were limited to five MeSH terms per token with varying weights applied. Run OHSU-5 was limited to 20 MeSH terms per token and was weighted at 1:2.

**Table 2.** Submission run characteristics

Run ID	Max Mesh Terms per token	MeSH Term Relative Weight (MeSH:Baseline)
OHSU-1	NA	NA
OHSU-2	5	1:01
OHSU-3	5	1:02
OHSU-4	5	1:05
OHSU-5	20	1:02

## Results

### Challenge results

**Figure 3.**
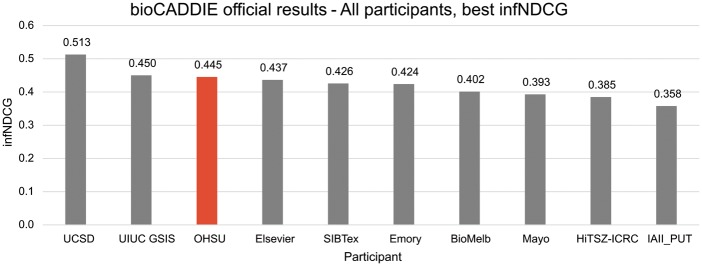
Official bioCADDIE challenge results—all participants, best infNDCG.

The official results for all OHSU runs are summarized in Table 3, and a comparison of the best runs of all challenge participants, based on infNDCG, is provided in Figure 3. At the time of publication, the official results are the only information we have regarding other groups’ participation in the challenge. Our highest scoring run, OHSU-4, used five MeSH terms with a relative weight of 1:5 to achieve an infNDCG of 0.4454 – the third highest in the challenge. When limited to the top ten documents retrieved, OHSU-4 achieved an NDCG@10 of 0.6122 and P@10-partial of 0.76. The baseline run, OHSU-1, did not score as high in infNDCG at 0.3965; however, when limited to top ten documents both NDCG@10 (0.6006) and P@10-partial (0.7467) scored comparably.

**Table 3.** Official OHSU bioCADDIE challenge results

Run ID	infAP	infNDCG	NDCG@10	P@10	P@10
				(+partial)	(−partial)
OHSU-1	0.3193	0.3965	**0.6006**	**0.7467**	0.3333
OHSU-2	0.1396	0.4024	0.3953	0.48	0.1933
OHSU-3	0.1921	0.4405	0.5345	0.6533	0.28
OHSU-4	0.2862	**0.4454**	**0.6122**	**0.76**	0.3333
OHSU-5	0.083	0.3156	0.2531	0.34	0.1133

Bolded scores emphasize high performance runs.

### Expanded results

**Figure 4.**
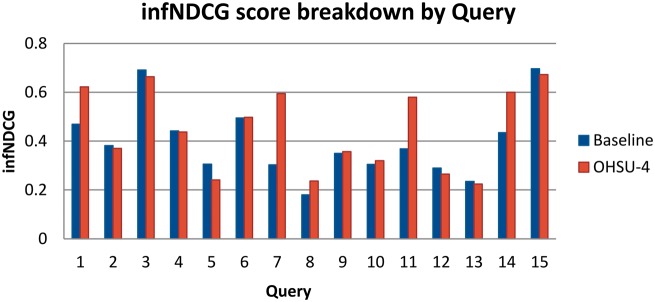
Score breakdown by query.

Using the resulting qrels file in conjunction with the treceval package, we performed score validation and further experimentation. First, we performed a breakdown of our scores by individual query using settings that match our best performing run, OHSU-4 as well as the baseline query. We found that scores vary considerably between queries for both the baseline as well as using query expansion. These results are shown in Figure 4. Compared with the baseline run, OHSU-4 performs 31–96% better in infNDCG in 5 of the 15 test queries (queries 1, 7, 8, 11 and 14), performs worse, at −21%, with only one query (query 5), and imparts little difference for the remaining queries.

**Figure 5.**
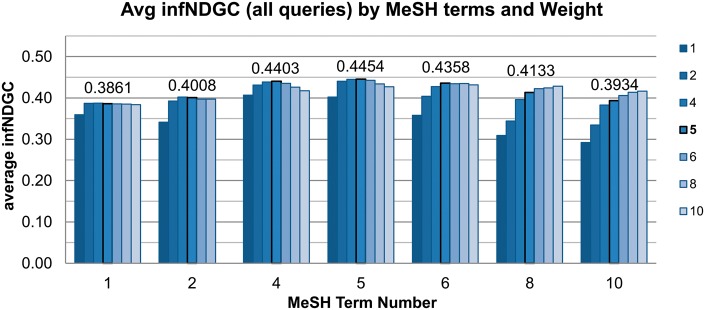
MeSH terms and Weight analysis.

We also performed an array of runs to determine the optimal number of MeSH terms and term weighting for our system. Figure 5 shows the average infNDCG across all queries for varying numbers of MeSH terms and weights. The settings used with run OHSU-4, with 5 MeSH terms at a 1:5 weighting, still achieves the best results when averaged over all queries.

Further analysis of individual queries with regard to optimal number of MeSH terms and weights reveals settings are highly variable depending on the query. Table 4 lists the best MeSH term number and weights for each query, along with the resulting theoretical best infNDCG for each query and the difference from the baseline infNDCG scores.

**Table 4.** Best Mesh Terms and Weight (Wt- Baseline:MeSH) per query compared with baseline score

Query	Baseline infNDCG	Best term	Best Wt	Theoretical Best infNDCG	Improvement over baseline
**1**	0.470	10	1:6	0.673	0.20
**2**	0.382	4	1:1	0.608	0.23
**3**	0.691	5	1:10	0.688	0.00
**4**	0.442	4	1:4	0.449	0.01
**5**	0.306	2	1:5	0.305	0.00
**6**	0.495	4	1:1	0.631	0.14
**7**	0.303	5	1:1	0.884	0.58
**8**	0.181	4	1:4	0.244	0.06
**9**	0.350	10	1:2	0.631	0.28
**10**	0.305	10	1:5	0.375	0.07
**11**	0.369	10	1:6	0.67	0.30
**12**	0.290	2	1:8	0.284	−0.01
**13**	0.235	10	1:1	0.243	0.01
**14**	0.435	5	1:6	0.611	0.18
**15**	0.696	1	1:1	0.746	0.05

## Discussion

### Key findings

Our results demonstrate that MeSH-based automatic query expansion and term re-weighting improve on our baseline search system to achieve scores that are comparable with other leading research groups in the bioCADDIE challenge. Our best performance in the official challenge results is achieved using query expansion limited to five MeSH terms per query token with a relatively light weighting, and yields an overall improvement in infNDCG of 11% over the baseline system. This is approximately near the middle of the range of improvement seen in other studies using vocabulary-based query expansion techniques (16,18,24).

Our attempts to fine-tune the number of MeSH terms and relative weights to improve system performance did not yield an improvement in overall scores. After extensive testing, we found that the number of MeSH terms and weights that achieve the best performance for our system was the same combination as initially chosen for our highest-scoring challenge submission run.

Performing additional analysis using the qrels file, we found that our scores between individual queries varied significantly—a phenomenon that is commonly seen in TREC evaluations (22,25). Compared with the baseline scores, the overall score improvement with query expansion can largely be attributed to higher scores seen in a handful of queries. Classically, query expansion performs well on average but can struggle greatly with some particular queries (15). Our system perhaps limits the negative components of query expansion through our use of the ‘should’ clause in Elasticsearch. By not requiring expansion terms in results, in addition to a lighter weighting against the baseline query, poorly matched MeSH terms may impart a smaller effect on results, yet the benefits from well-matched synonyms are still applied.

We also found that the best number of MeSH terms and weights to use varies significantly between queries, although for many queries, a range of settings achieves results similar to the best. If our system were theoretically able to select the optimal number of MeSH terms and weighting to use for each query, as demonstrated in Table 4, the application of MeSH term-based query expansion would not perform >2% worse than the baseline system for any query and would result in > 10% score improvement for 9 of the 15 queries.

While intriguing, these findings can be difficult to interpret and there are many complexities to consider. First, as we use the ‘should’ clause for expansion terms, using a very light weighting is nearly equivalent to not using query expansion at all. Second, the total number of MeSH terms returned for each token may not be equal to the set cap and thus not be the actual number used for expansion. Third, the terms returned from the Entrez service for many queries appear to be irrelevant. Finally, the variable with the largest effect on scores are the queries themselves.

Nevertheless, these data suggest the key to gaining the full benefit from query expansion techniques partly lies within a system’s ability to either predict which queries would benefit from expansion and selectively apply query expansion to those queries, or even predict the best relative weighting to use for each query. This prediction could be based on many factors such as query length or perhaps some estimate of the quality of synonym terms. A prediction algorithm could even be trained by using judged challenge databases like bioCADDIE; however, due to the high variability between queries, it is likely many more queries would need to be judged for relevance before this could yield satisfactory results.

### Limitations and implications for further work

Numerous improvements to our methods could be implemented that would likely further improve system performance. For example, many advanced techniques have been described for obtaining and determining relevance of synonym terms (16,18,21,24,33,34). One such method, utilizing a graph database such as Neo4j (http://neo4j.org/), could allow for direct mapping of MeSH terms with associated clinical constructs (35). Using this approach, query expansion using only MeSH terms that are directly related to an identified construct such as disease or species could be effectively implemented.

Our system does not directly address the ambiguity problem of lexically related terms and simply includes all fetched synonyms in the MeSH query. The system somewhat compensates by use of the ‘should’ clause in Elasticsearch. This may limit the negative impact that unrelated MeSH terms have and allow the more relevant synonyms from other query terms to shape the final results. Additionally, by limiting synonyms to the curated MeSH database we increase the initial likelihood of finding relevant terms. However, these methods could likely be improved by implementing a system to filter or independently weigh synonym terms based on a statistical relationship model (36).

We did not attempt to fully utilize the structured metadata of the bioCADDIE dataset and instead our system essentially treated all fields as unstructured text data. While our automatic import process identified 128 distinct metadata fields, a major limitation of the process is that nested arrays were imported as flattened objects (37). The fields in these objects were still searchable as text, but the relationships between fields within each object are lost. This, in addition to the fact that numerous fields were utilized by only a sparse number of individual datasets led us to focus efforts elsewhere. However, consistent and standardized metadata fields could be very powerful in enhancing search of this nature, and further studies to evaluate novel methods of metadata use should be performed.

While the Elasticsearch Standard Analyzer performs tokenization of all database fields, our initial parsing with regular expression matching and use of NLP with the NLTK only yields basic tokenization of the input query. More advanced NLTK tools could be used to tag query tokens with one or more metadata types, such as grammatical type, data type or score such as uniqueness. Metadata from tagged tokens could then be used for database filtering, such as for a specific data type, disease state or species. Metadata from tags could also be used to improve query expansion, e.g. by limiting expansion to nouns or the most unique terms, or by filtering the related MeSH terms in a similar fashion as above. Additionally, the NLM’s MetaMap tool could be used to perform tokenization of the input query. MetaMap has many advantages including mapping input queries directly to the Unified Medical Language System (UMLS). Concepts which would allow far greater control over the selection of query expansion terms (38). More advanced systems have successfully used MetaMap in this fashion (33).

Evaluation of variables such as optimal number of MeSH terms and weighting after challenge completion using the resulting qrels file has many limitations. This challenge evaluated only 15 queries and the qrels file only contained relevance judgments from a sampling of documents from submitted runs (30). Any novel documents returned from additional runs would not have an associated relevance judgment and thus should not result in an increased score. Further, it is possible that additional runs may be more likely to retrieve documents using similar methods as submitted challenge runs, resulting in artificially higher scores. Efforts to test more queries and judge more documents for relevance would enhance the reliability of the bioCADDIE challenge dataset as an IR testbed and reduce bias introduced by the specifics of any particular query or search method.

Another potential area for improvement involves the analysis and storage of the bioCADDIE database. While Elasticsearch’s default import and analysis methods allow establishing a working search engine quickly, using Elasticsearch as a primary database has many limitations. Storing the working database in a separate relational or graph database and using a connection to the Elasticsearch cluster would allow more control over the analysis and use of metadata stored in bioCADDIE. This would more easily allow the correct mapping of all existing metadata fields as well as techniques to clean the database, such as joining metadata fields that contain the same data but are of a different name. This would also support the creation of entirely new metadata fields that could be populated from various techniques, such as the analysis of bioCADDIE’s free-text data fields or even by scraping online databases such as PubMed.

## Conclusion

Our submission to the 2016 bioCADDIE search retrieval challenge showed that MeSH term-based query expansion can be used to enhance search retrieval of metadata for biomedical datasets. Further testing demonstrates that are appropriate MeSH term number and weight selection is important for the best query expansion results. While our system has significant room for improvement, we were able to achieve competitive results without the use of complex techniques such as machine learning algorithms. Future experiments would benefit from more test queries and relevance judgments, advanced NLP and query expansion techniques, custom database designs and better utilization of Elasticsearch tools to take advantage of structured metadata fields.

## Funding

Funding for this publication was provided by the OHSU Department of Medical Informatics and Clinical Epidemiology. bioCADDIE Dataset Retrieval Challenge was supported by the NIH Grant U24AI117966.

*Conflict of interest*. None declared.
